# Supplementary material for: New Insight into Muscle-Type Cofilin (CFL2) as an Essential Mediator in Promoting Myogenic Differentiation in Cattle
Source: Bioengineering (Basel). 2022 Nov 25;9(12):729. doi: 10.3390/bioengineering9120729 (PMC9774386; doi:10.3390/bioengineering9120729)
Supplement: Supplementary file 1 [file bioengineering-09-00729-s001.zip › bioengineering-2039564-supplementary.pdf]

Supplementary Data: Overexpression or Knock-down Vector Construction of CFL2

We designed and synthesized *CFL2* overexpression primers containing *Kpn*I and *Hin*dIII (TaKaRa, Dalian, China) restriction sites, the coding sequences of *CFL2* were subcloned into the pAdTrack-CMV (Miaoling bio, Wuhan, China) plasmid vector to construct the recombinant shuttle vector pAdTrack/CMV-CFL2. Then, this vector was homologously recombined with plasmid pAdEasy-1 to generate an adenoviral plasmid in BJ5183 (Miaoling bio, Wuhan, China) cells. The adenoviral plasmids linearized by *Pac*I (TaKaRa, Dalian, China) were transfected into 293A cells to generate the adenovirus pAdEasy-1/pAdtrack-CMV-CFL2 (CFL2-CMV).

The BlockiT shRNA interference system was used to design and synthesize two pairs of primers for the *CFL2* gene (shCFL2-1F, shCFL2-1R, shCFL2-2F, shCFL2-2R) and one pair of primers for the negative control (NC, shRNA-NC-F and shRNA-NC-R). All primer sequences containing *Bam*HI and *Hin*dIII (TaKaRa, Dalian, China) restriction sites. The oligonucleotides were cloned into the pENTR/CMV-GFP/U6 vector (Miaoling bio, Wuhan, China) to construct shuttle vectors pENTR/CMV-GFP/U6-shCFL2-1 and pENTR/CMV-GFP/U6-shCFL2-2 and then recombined with the adenovirus backbone vector pAD/PL-DEST (Miaoling bio, Wuhan, China) to produce recombinant vectors pAD/PL-DEST/CMV-GFP/U6-shCFL2-1 (shCFL2-1) and pAD/PL-DEST/CMV-GFP/U6-shCFL2-2 (shCFL2-2).

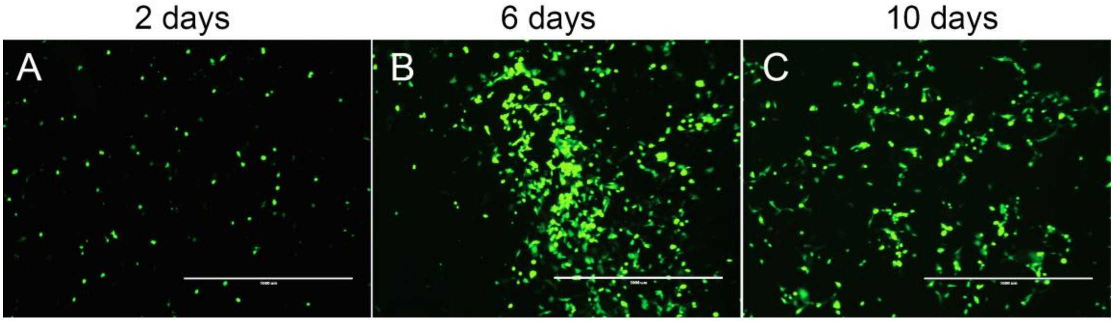

Figure S1. Production of CFL2-CMV recombinant adenovirus in 293A cells. *Pac* I-digested pAdEasy-GFP-CFL2 was transfected into 293A cells and GFP expression was visualized by fluorescence microscopy at the indicated times thereafter. Comet-like adenovirus-producing foci became apparent after 6 days. 80% cells became round and could receive virus after 10 days.

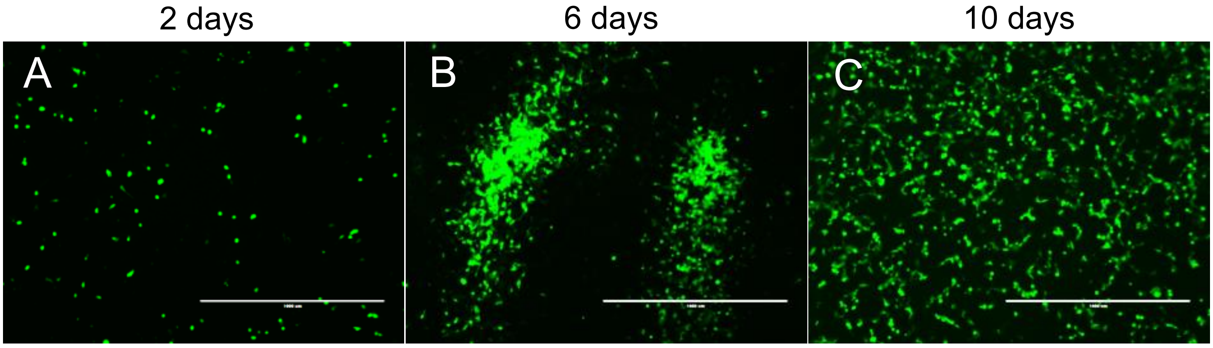

Figure S2. Production of shCFL2-1 recombinant adenovirus in 293A cells. *Pac* I-digested pAdEasy-GFP-shCFL2-2 was transfected into 293A cells and GFP expression was visualized by fluorescence microscopy at the indicated times thereafter. Comet-like adenovirus-producing foci became apparent after 6 days. 80% cells became round and could receive virus after 10 days.

Supplementary Data: The published miRNAs to skeletal muscle myoblast development in cattle

Table S1. The published miRNAs to skeletal muscle myoblast development in cattle

Name	Tatget gene	Associated phenotype	References PMID
Bta-miR-1	HDAC4/LOC536229	Skeletal muscle satellite cell myogenic differentiation	26424132
Bta-miR-107	Wnt3a	Suppress cell differentiation and did not affect cell proliferation	29858062
Bta-miR-125b	IGF2	Sponged by lncMD to promote myoblast differentiation	27589905
Bta-miR-182	CFL1	Promoted myoblast differentiation	35627108
Bta-miR-206	HDAC4/LOC536229	Skeletal muscle satellite cell myogenic differentiation	26424132
Bta-miR-365-3p	AVCR1	Promoted myoblast differentiation and inhibited proliferation	33431058
Bta-miR-378a-3p	HDAC4	Promoted myoblast differentiation and inhibited proliferation	27661135
Bta-miR-744	Wnt5a/CaMKIIδ	Promoted skeletal muscle cell proliferation while inhibited differentiation	31051333
Bta-miR-885	MYOD1	Promote proliferation but inhibit differentiation	331985035

Supplementary Data: Western blot and COBRA

Total proteins were extracted from cells using radio immunoprecipitation assay (RIPA) lysis buffer containing 1 mM phenylmethylsulfnoyl fluoride (PMSF) (Solarbio, Beijing, China). Proteins were measured and adjusted by using the BCA protein assay kit (MultiScience, Hangzhou, China) and denatured with 5×SDS loading buffer (Beyotime, Shanghai, China) at 98 °C for 10 min.

The COBRA (Combined bisulfite restriction analysis) technique is a variation of bisulfite sequencing and combines bisulfite conversion-based polymerase chain reaction with restriction digestion. The PCR products hold a natural *Hin*fI endonuclease restriction site (G^ANTC) when the CpG dinucleotides are methylated; otherwise, the site cannot be digested if one or more CpG dinucleotides within its recognition sequence are unmethylated. During sodium bisulfite treatment, unmethylated cytosine residues were converted to "T", whereas methylated cytosine residues were retained as "C". Therefore, in the mixed population of resulting PCR fragments, the ratio of band intensity of digested fraction to the combined intensities of both digested and undigested fractions reflected the levels of DNA methylation on the restriction sites.

Supplementary Data: Identification of bovine primary myoblast cells

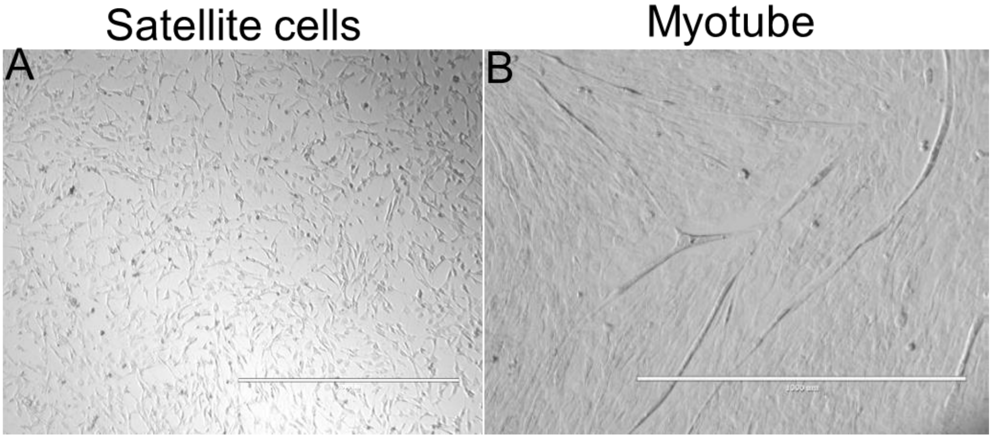

Figure S3. Identification of bovine primary myoblast cells (×100). (A*)* Bovine primary myoblast cells cultered from bovine longissimus muscle. (B) The cells on the day 5 of induced differentiation.
